# Circularly Polarized S Band Dual Frequency Square Patch Antenna Using Glass Microfiber Reinforced PTFE Composite

**DOI:** 10.1155/2014/345190

**Published:** 2014-05-22

**Authors:** M. Samsuzzaman, M. T. Islam, Haslina Arshad, J. S. Mandeep, N. Misran

**Affiliations:** ^1^Department of Electrical, Electronic and Systems Engineering, Faculty of Engineering and Built Environment, Universiti Kebangsaan, 43600 Bangi, Selangor, Malaysia; ^2^Centre of Artificial Intelligence Technology, Faculty of Information Science and Technology, Universiti Kebangsaan, 43600 Bangi, Malaysia

## Abstract

Circularly polarized (CP) dual frequency cross-shaped slotted patch antenna on 1.575 mm thick glass microfiber reinforced polytetrafluoroethylene (PTFE) composite material substrate is designed and fabricated for satellite applications. Asymmetric cross-shaped slots are embedded in the middle of the square patch for CP radiation and four hexagonal slots are etched on the four sides of the square patch for desired dual frequency. Different substrate materials have been analysed to achieve the desired operating band. The experimental results show that the impedance bandwidth is approximately 30 MHz (2.16 GHz to 2.19 GHz) for lower band and 40 MHz (3.29 GHz to 3.33 GHz) for higher band with an average peak gain of 6.59 dBiC and 5.52 dBiC, respectively. Several optimizations are performed to obtain the values of the antenna physical parameters. Moreover, the proposed antenna possesses compactness, light weight, simplicity, low cost, and circularly polarized. It is an attractive candidate for dual band satellite antennas where lower band can be used for uplink and upper band can be used for downlink.

## 1. Introduction

Modern small satellites allow for the achievement of many tasks and experiments in space. Nowadays, miniaturized technology makes it feasible to build small satellites. All of the subsystems constituting a small satellite must be designed to respect severe physical limitations and restrictions. The dimensions of the mini- and microsatellites generally render reflector antennas inadequate, even if they are small [1, 2]. In addition, the placement of mechanical elements, needed to deploy such antennas after reaching space, is a significant problem. On the other hand, helical antennas were widely used in traditional spacecrafts because of their wide beams and circular polarization. However, they have become unsuitable for minisatellites [3]. The protrusion of such an antenna, on a retractable boom, requires mechanical elements that are especially susceptible to mechanical failure. Insatiable antennas, despite their large dimensions, do not need protrusion mechanisms, but the technology is still in its infancy. Additionally, the deployment of a large bowl may easily block solar energy. For these reasons, the type of on-board antennas for small spacecrafts should be investigated very carefully. The main feature of minisatellite antennas is the lightweight structure and the high degree of integration. In small satellites, it is preferred to use antennas placed on outer walls, which may be easily bonded with a thermal blanket. Among the different types of low profile antennas, microstrip antennas have gained the greatest interest. Single-feed microstrip patch antennas using various types of slots have been investigated to produce CP rectangular patch antennas [4–10]. In [4], the authors proposed a dual feed circular polarization antenna for RFID application with size of 130 mm × 104 mm. A single-feed annular slot CP antenna was presented in [5]. However, the antenna has 50 mm × 50 mm dimension and achieved high frequency axial ratio bandwidth. To obtain different radiation characteristics at two operating frequencies, [6] proposed a stacked patch antennas for GPS and WLAN applications. In [7], small frequency ratios about 1 : 1.1 were obtained by an unequal cross slot embedded in the circular patch and two orthogonal linear stubs spurred from the annular ring. With this configuration, the proposed antenna achieved only 0.4 dBi gain. Another dual band stub loaded CP antenna [8] was proposed with dimension of 100 mm × 100 mm and used four short circuit walls. As a result, the design has been complicated. In [9, 10], the authors proposed a square patch with centre slot and rectangular patch with ground plane ring slot CP antenna. But square patch designs only realize one CP radiation. On the other hand, rectangular patch with ground plane ring slot CP antenna achieved dual frequency but no gain was mentioned in this paper [10]. Modified dual band stacked patch CP antenna was designed for GPS application [11]. In this paper, a three-layer dual feed stacked microstrip antenna was designed with thickness more than half a wavelength. Nowadays, there are more critical requirements for the antennas such as dual band operation, better low angle radiation pattern, and improved circular polarization level, which are even more difficult to realize. Many methods were used to widen beams and enhance the gain at low angle, for example, using high permittivity substrates, operating at higher-order modes, and using four circular slot ring arrays [12, 13].

On the other hand antenna miniaturization obtains a significant amount research attention towards the contemporary wireless communication development for the past decade. Several antenna miniaturization techniques have extensively been studied by a number of researchers such as using slotted radiating patch, high dielectric material substrate [14–16], artificial magnetic conductor [17, 18], electromagnetic band-gap (EBG) structure [19], metamaterial [20], and magnetodielectric materials [21]. One of the most effective antenna miniaturization techniques is using dielectric composite material as a substrate. The overall size of the antenna can be significantly reduced by using glass microfiber reinforced PTFE composite material substrate without negotiating the overall performance due to high relative permittivity and low loss. In the literature a considerable number of academicians and professionals have comprehensively examined the use of high dielectric ceramic materials as substrates for miniature antenna design. A genetic antenna was designed with dimension of 25 mm × 33 mm × 30 mm. It was immersed in a dielectric powder over a 120 mm × 120 mm ground plane in a 48 mm conical cup for multifrequency application [22]. A 24 GHz mixed low temperature cofired ceramic (LTCC) fractal antenna array with the dimension of 24 mm × 24 mm × 4.8 mm was presented [23]. A 60 mm × 60 mm LTCC antenna on EBG structure is proposed for the European upcoming GALILEO satellite [24]. However, one still needs to put more research effort onto antenna miniaturization with improvement of bandwidth, gain, efficiency, and so forth.

In this paper, a 40 mm × 40 mm square cross-shaped slotted patch antenna fed by a coaxial probe has been designed and printed on 1.575 mm thick glass microfiber reinforced PTFE composite material substrate with relative permittivity of 2.33 and a tangent loss of 0.0012. The −10 dB return loss bandwidth ranges from 2.16 GHz to 2.19 GHz (30 MHz) and from 3.29 GHz to 3.33 GHz (40 MHz) are measured. Gains of 6.59 dB and 5.52 dB at 2.18 MHz and 3.30 GHz, respectively, are achieved. The main advantage of CP versus linear polarization is that CP eliminates polarization mismatch losses caused by Faraday's rotation and varies the squint angle of polarization vectors between stations on the earth. The overall size of the antenna is reduced by using glass microfiber reinforced PTFE composite material.

## 2. CP Slotted Patch Structure Design Methodology Technique

**Figure 1:**
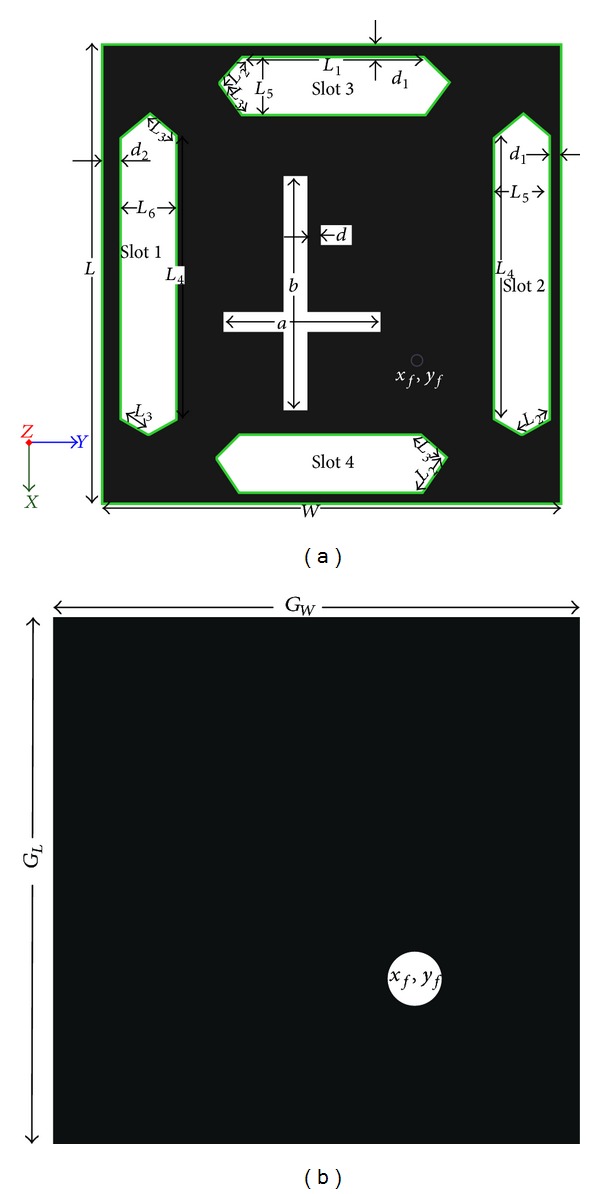
Proposed antenna geometry layout: (a) top view and (b) bottom view.

The dual frequency antenna is suitable for using up- and downlink to miniaturize the size. In addition, the transmission wave can be circularly polarized to minimize the effects that craft rotation could have on a linearly polarized. Initially a square patch is designed with a slot for achieving dual frequency and then central two slots are introduced to obtain circular polarization as shown in Figure 1. The central slot is perpendicular to each other and one is greater than the other. Central slot initial design equations are as follows [25]:

(1) $$\begin{array}{c} c=\frac{L}{2.72}=\frac{W}{2.72},\\ d=\frac{c}{10}=\frac{L}{2.72}=\frac{W}{2.72}, \end{array}$$

where *L* and *W* are the length and width of the patch, *c* is the length of slot, and *d* is the width of the slot.

The dual frequency operation of the slatted structure can be interpreted as two modes that arise from the perturbation of the TM_100_ and TM_300,_ respectively. Simple semiempirical formulas, based on physical models, can be used [26]:

(2) $$\begin{array}{c} f_{100}=\frac{c}{2\left(W+\Delta W\right)\sqrt{\in_{e}\left(L/t,\in_{r}\right)}}G,\\ f_{300}=\frac{c}{2\left(L-2l+d\right)\sqrt{\in_{e}\left(L/t,\in_{r}\right)}}, \end{array}$$

where *c* is the free space of light and *Z* is the velocity of light in free space. Consider

(3) G=1.13−0.19LsW−0.73wW,ΔW=tπL/t+0.336L/t+0.556 ×{0.28+∈r+1∈r[0.274+ln⁡(Lt+2.518)]}.

The location of the slots with respect to the patch is defined by the dimensions *w* and *l*.

Figure 1(a) shows the geometric layout of a proposed single-feed circularly polarized slotted rectangular microstrip patch antenna. Two asymmetric pairs of hexagonal slots are etched on the four sides of the patch. Slots 3 and 4 are identical with different arm lengths of *L*_1_, *L*_2_, and *L*_3_ and a slot width length of *L*_5_. On the other hand, slot 1 and slot 2 are not identical, but arm length *L*_4_ is the same. The distance from the sides of slots 2, 3, and 4 is *d*_1_. An asymmetric cross-shaped slot, (a), (b), is placed in the centre of the square patch radiator for CP radiation and good impedance matching. The single coaxial probe is located along the orthogonal axis of the square patch radiator. The coaxial feed location (*x*_*f*_, *y*_*f*_) is on the *x*-axis. The proposed antenna is designed on a microwave Rogers/RT Duroid substrate of thickness *t* and relative permittivity of 2.33. All of the structural parameters of the proposed antenna are listed in Table 1.

## 3. Results and Discussion

### 3.1. Parametric Study of the Proposed Antenna

**Figure 2:**
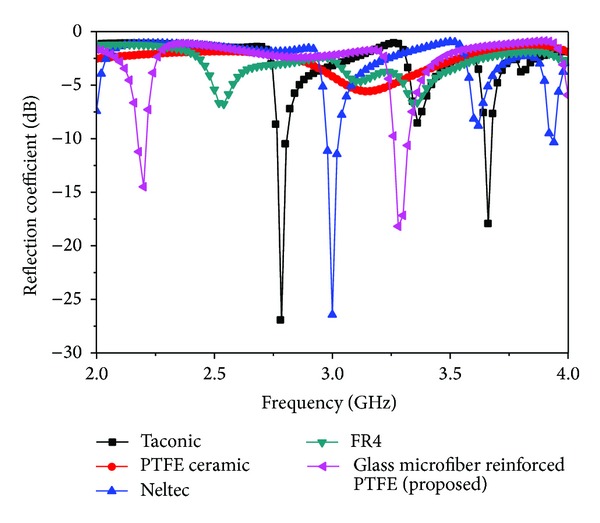
Reflection coefficient for different substrate materials.

A parametric study has been conducted to analyse the effect of different dielectric material substrates and to optimize the one of the most important different parameters of the proposed antenna. The analysis helps to investigate the effects of the different parameters on impedance bandwidth. The dielectric properties of the different materials are tabulated in Table 2. The effects of different substrate materials on the reflection coefficient of the proposed antenna are shown in Figure 2. It can be clearly stated that the proposed material antenna provides the desired frequency and acceptable return loss values compared to the other stated materials. The antenna with the Neltec material substrate produces a lower return loss value and a single resonance. Taconic produces dual resonance frequencies but a low return loss value. Actually RT/Duroid 5870 consists of glass microfiber reinforced PTFE composites which are designed for exacting strip line and microstrip circuit applications. Glass reinforcing microfibers are randomly oriented to maximize benefits of fiber reinforcement in the directions most valuable to circuit producers and in the final circuit application. The dielectric constant of RT/Duroid 5870 laminates is uniform from panel to panel and is constant over a wide frequency range. Its low dissipation factor extends the usefulness of these laminates to different satellite applications. It is easily cut, sheared, and machined to shape. They are resistant to all solvents and reagents, hot or cold, normally used in etching printed circuits or in plating edges and holes.

**Figure 3:**
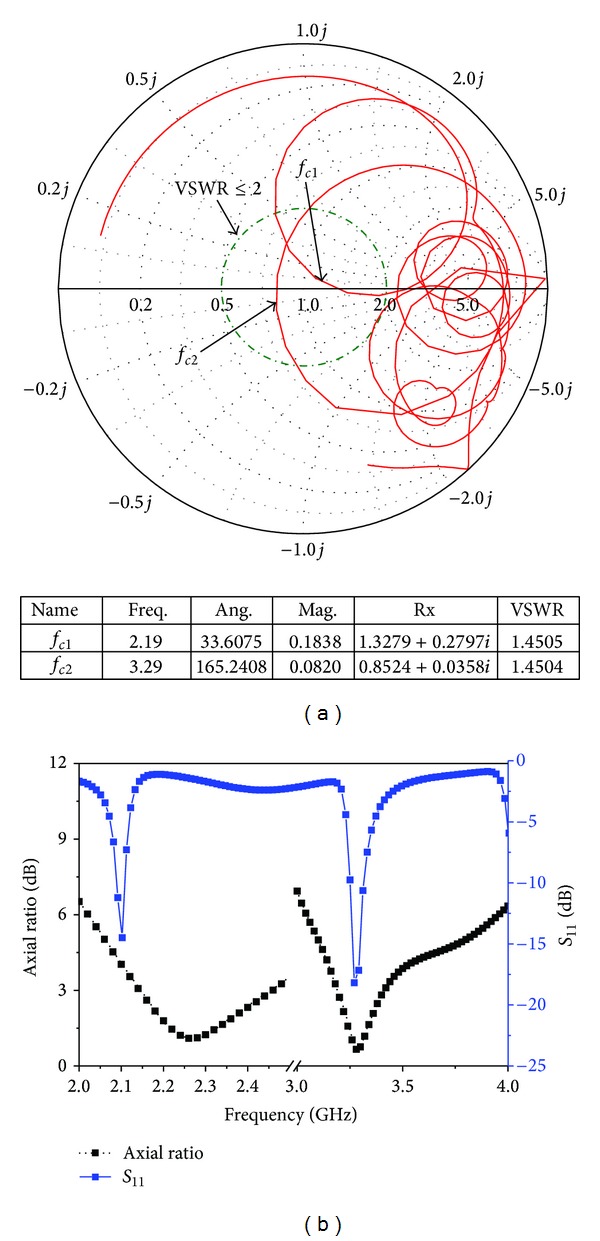
Simulated results for the proposed antenna with optimized parameter Table 1. (a) Smith chart and (b) reflection coefficient and axial ratio.

The antenna was simulated using a three-dimensional electromagnetic finite element method (FEM) based simulator HFSS. In the design process, several major parameters are examined: patch length *L*, width *W*, cross-shaped length *a*, bandwidth *d*, and feed position *x*_*f*_, *y*_*f*_. The electromagnetic wave propagates in the projected direction, regulated by the parasitic resonator element on the top, which simultaneously acts as an impedance matching element. Microstrip radiating patch element design involves estimating its dimensions. The frequency bands depend on the length of the radiating element and the slot location for the lower frequency band and on the feeding position and microstrip line characteristics of the higher frequency band. The dimension of the radiating patch element is abridged by introducing the slots into the conventional rectangular patch. The current flowing in the radiating patch proceeds along the longer patch and around the slots, until it touches the opposite edge. By introducing cutting slots into the rectangular patch, the desired resonant band is achieved and circular polarization is obtained using two cross-shaped slots.  Figure 3 shows the simulated input impedance, reflection coefficient, and axial ratio of the proposed slotted antenna using the optimized parameter. It can be observed that the input impedance is close to 50 Ω with variations at 33 Ω and to 57 Ω at 2.17 and 2.21 GHz, respectively, while the magnitude of the imaginary components varies from −11.6 Ω at 2.17 GHz to 16.7 Ω at 2.21 GHz. Therefore, the proposed antenna element exhibits a low reflection coefficient less than −10 dB over the entire operating band (2.17–2.21 GHz, 3.27–3.32 GHz). Axial ratio bandwidths of less than 3 dB are also achieved for ranges between 2.12 to 2.20 GHz and 3.27 to 3.30 GHz. Generally, the axial ratio is considered to determine antenna polarization. Antennas are circularly polarized if the value of the axial ratio is less than 3 dB, and for an ideal circularly polarized antenna the axial ratio should be 0 dB. It can be clearly stated that the value of the axial ratio is less than 3 dB in the desired operating band, which means that the proposed antenna can be considered to be circularly polarized.

**Figure 4:**
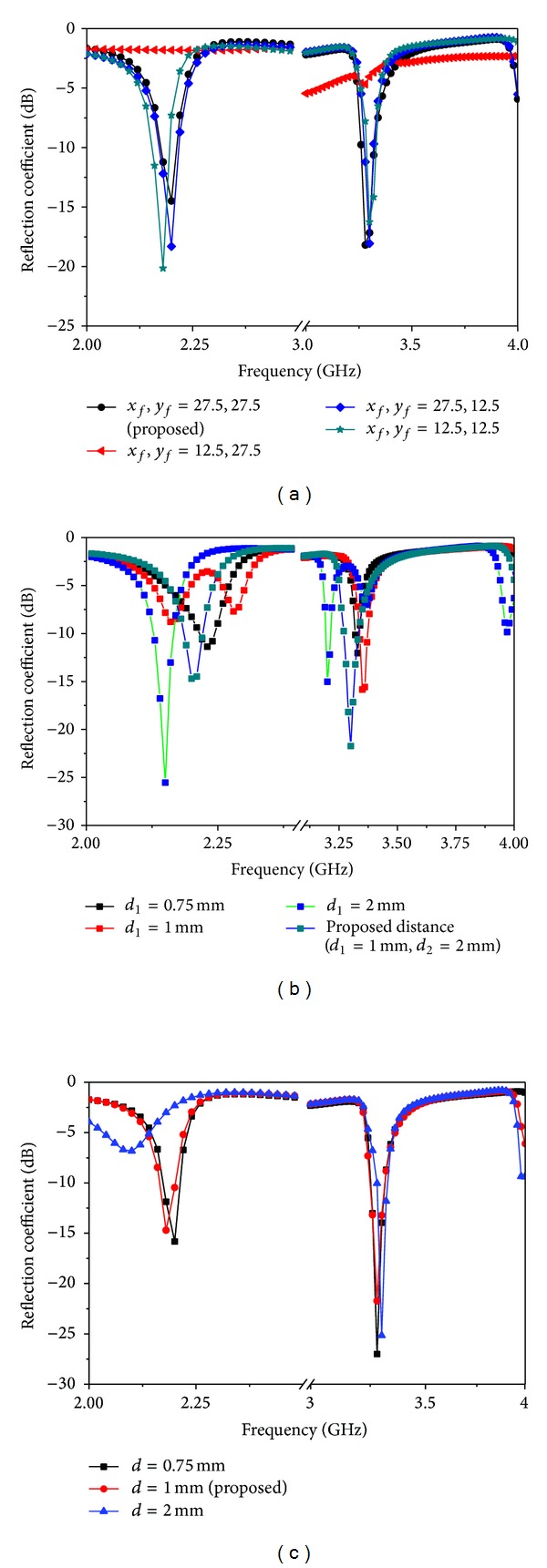
Reflection coefficient performance for the cross-shaped antenna for different values of (a) feed position (*x*_*f*_, *y*_*f*_) and (b) *d*_1_ and (c) *d*.

Figure 4(a) shows the effects while varying the feed position *x*_*f*_, *y*_*f*_ on the reflection coefficient. It is found that the proposed position is better than others based on the desired resonance frequency and impedance bandwidth.  Figure 4(b) shows the effect on the reflection coefficients of the various slot distances *d*_1_ from the four sides. It can be clearly stated that the proposed distance (left *d*_2_ = 2 mm, right, up, and down *d*_1_ = 1 mm) is the best distance based on the impedance bandwidth and desired frequency.  Figure 4(c) shows the reflection coefficients for the two slits, (a), (b), with width *d*.

**Figure 5:**
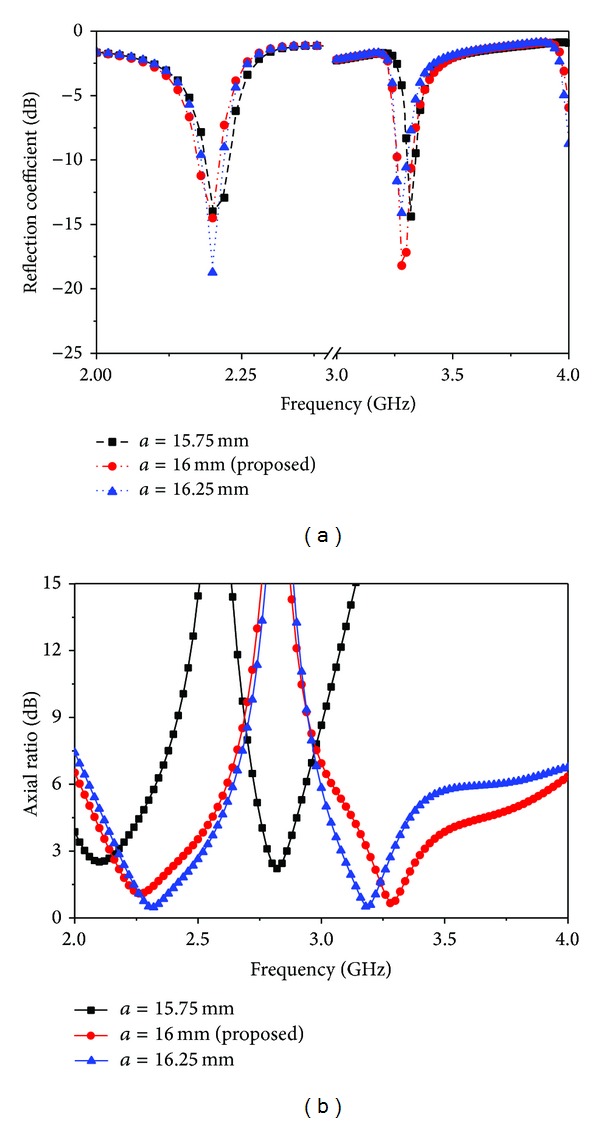
Different length reflection coefficient and axial ratio results for the cross-shaped slot antenna. (a) Reflection coefficient and (b) axial ratio.

**Figure 6:**
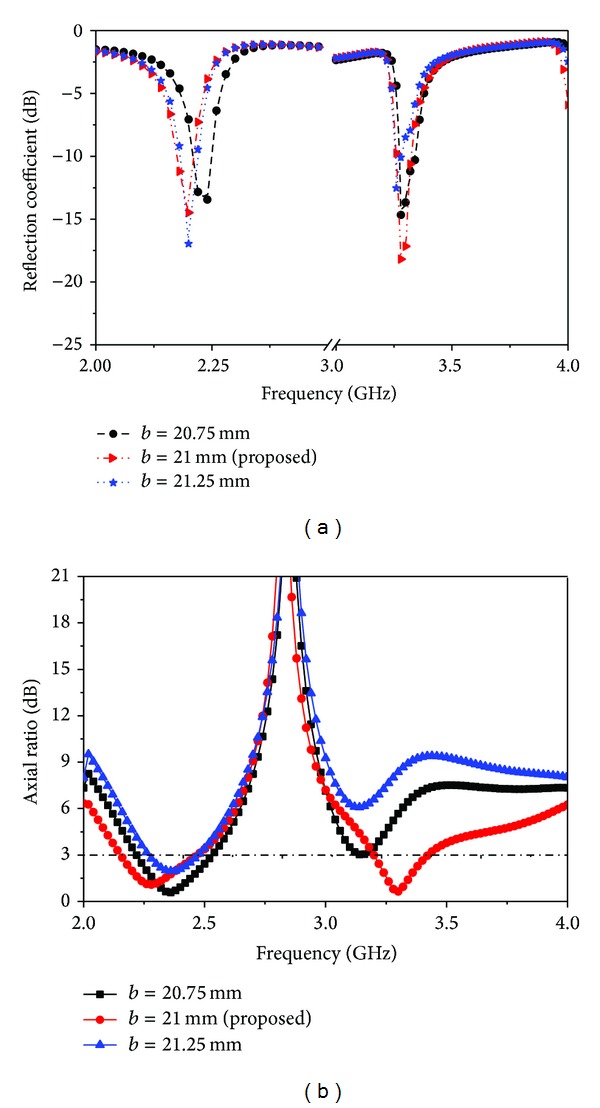
Different lengths of reflection coefficient and axial ratio results for the cross-shaped slot antenna. (a) Reflection coefficient and (b) axial ratio.

The simulated return loss and axial ratio are shown in Figure 5 for different values of the length of the cross-shaped slot *a*. It can be clearly observed that as *a* increases the lower band centre frequency remains at the same position, but the axial ratio is shifted, while the upper band resonance value decreases and the axial ratio is also shifted. On the other hand, when *a* is decreasing, the centre frequencies of the lower and upper bands and the upper band of the return loss and axial ratio will all decrease. It is also found that the performance of the upper band becomes much worse than that of the lower band when *a* diverges from its optimized value.  Figure 6 depicts the performance of the proposed antenna when the length (*b*) of the cross-shaped slot is varied. It can be observed that if the value of *b* is increased from its optimized value, then the lower resonance is shifted, but the upper resonance has no effect. On the other hand, the lower band axial ratio increases when *b* decreases, but the upper band axial ratio decreases. Again, in both of the operating bands, the axial ratio bandwidth is decreased when *b* is increased.

**Figure 7:**
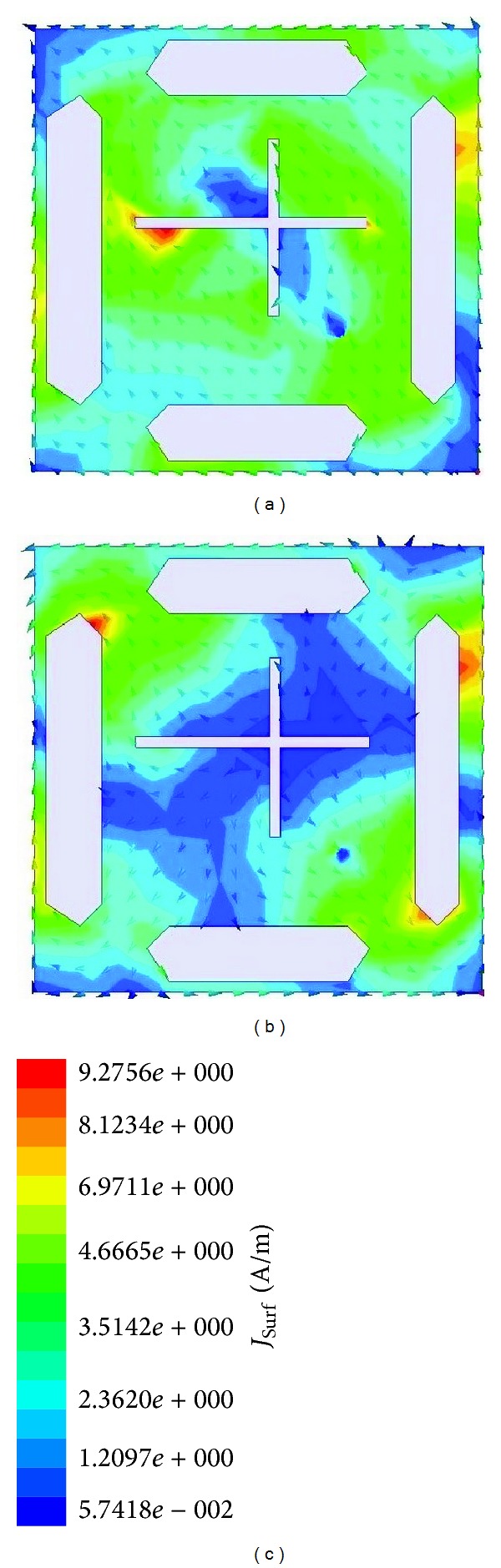
Surface current distributions at (a) 2.18 GHz, (b) 3.30 in phase 0° (c) scale.

In order to visualize the operation of the proposed antenna, the magnetic current concentrations of the aperture were simulated to investigate the generation of circular polarization within operating band.  Figure 7 demonstrates the current distribution which is observed from the positive *z*-direction at 2.18 GHz and 3.30 GHz when the phase angle is 0°. Note that two cross-shaped slots where *a* > *b*, these two rectangular slots move the direction of the current circular.

**Figure 8:**
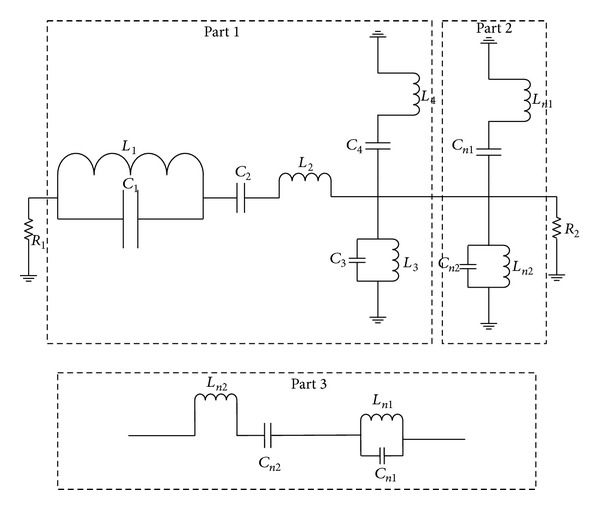
Equivalent circuit of the proposed patch antenna.

Figure 8 shows the equivalent dual band filter circuit of the proposed antenna. When *n* is even the circuit in PART 2 is attached to the circuit in PART 1 in serial connection to demonstrate the even mode of the antenna equivalent filter specified.

### 3.2. Prototype Development and Experimental Validation

**Figure 9:**
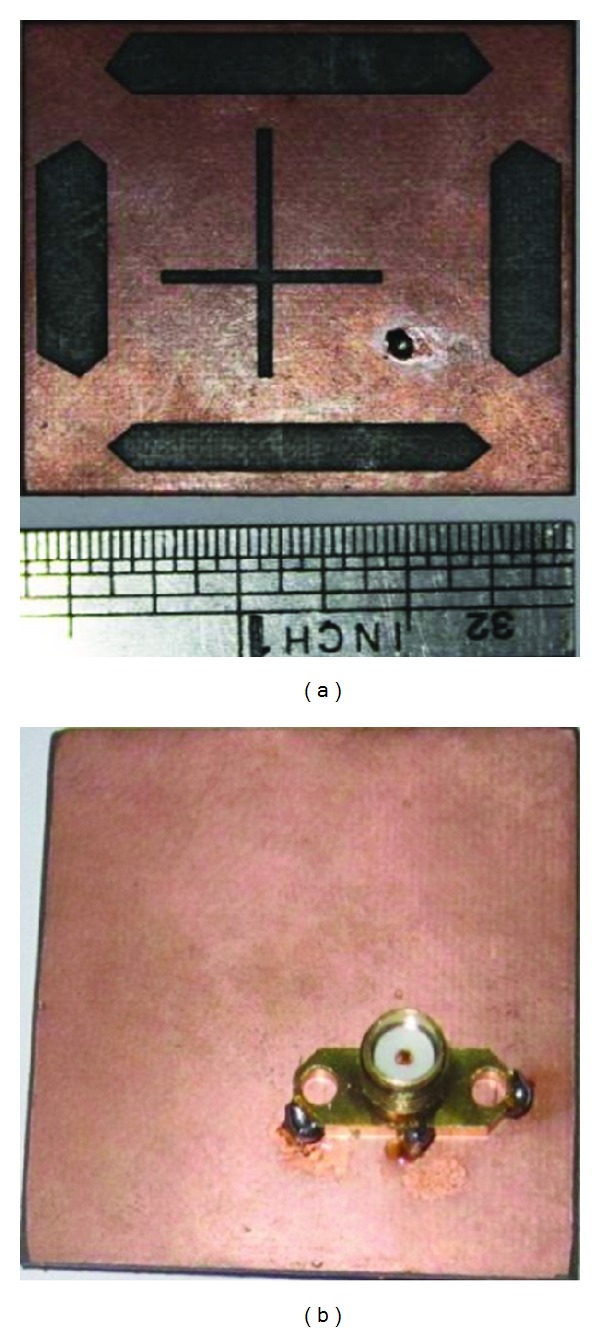
Prototype of the proposed antenna: (a) front view and (b) back view.

**Figure 10:**
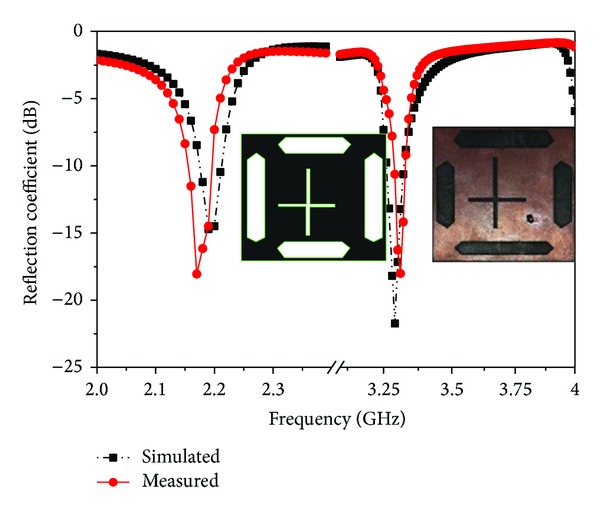
Comparison between simulated and measured return losses of the proposed antenna.

Figure 9 shows the photograph of the proposed prototype. The simulated and measured return losses of the proposed antenna are presented in Figure 10, and good agreement was established between the two results. In this figure, the −10 dB impedance of both frequencies centered at 2.18 GHz and 3.30 GHz, respectively, was measured to be 30 MHz and 40 MHz, respectively, confirming that the proposed antenna covers the S operating bands. The discrepancy is due to tolerances in the dielectric constant and loss tangent of the laminate, in addition to the soldering effect.

**Figure 11:**
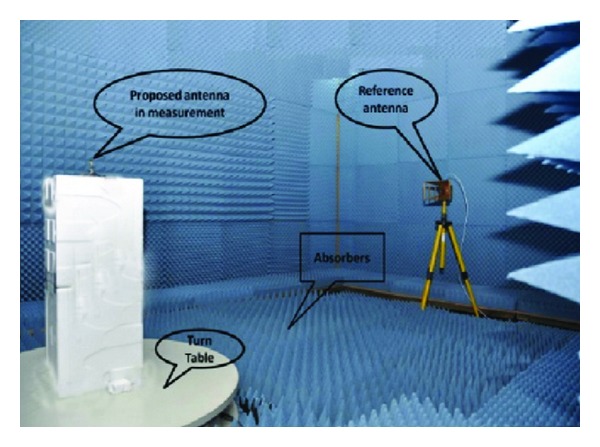
Anechoic chamber.

The radiation pattern of the proposed antenna was measured in a rectangular-shaped anechoic chamber. A double ridge guide horn antenna (SAS-571 from AH System Inc.) was used as reference antenna. The measuring antenna was placed face to face with the reference antenna. The photograph of the anechoic measurement chamber has been shown in Figure 11. Pyramidal shape electrically thick foam absorber has been used on the wall, ceiling, and floor with less than −60 dB reflectivity at normal incidence. A turn table of 1.2 m diameter has been used to rotate the measuring antenna with specification, 1 RPM rotation speed, 360° rotation angle connected with a 10-meter cable between controllers. Agilent vector network analyzers (VNA E8362C) ranges up to 20 GHz have been used for measurement procedure.

**Figure 12:**
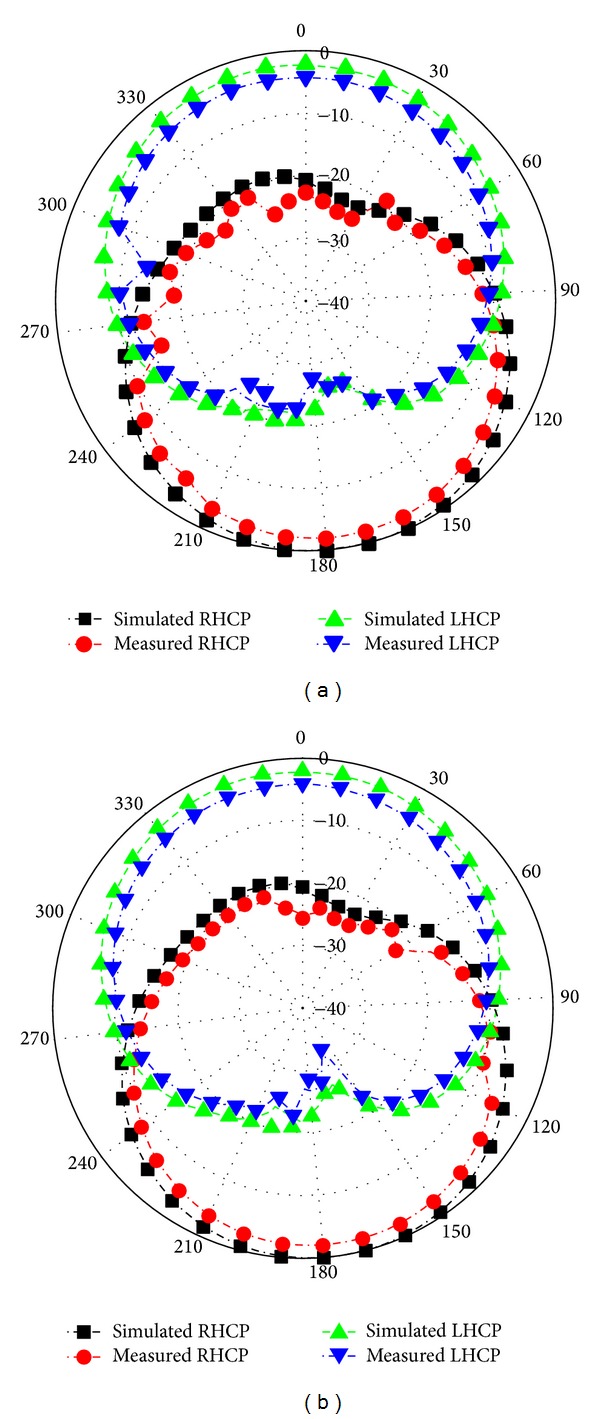
Measured and simulated radiation patterns at (a) 2.18 GHz and (b) 3.30 GHz.

**Figure 13:**
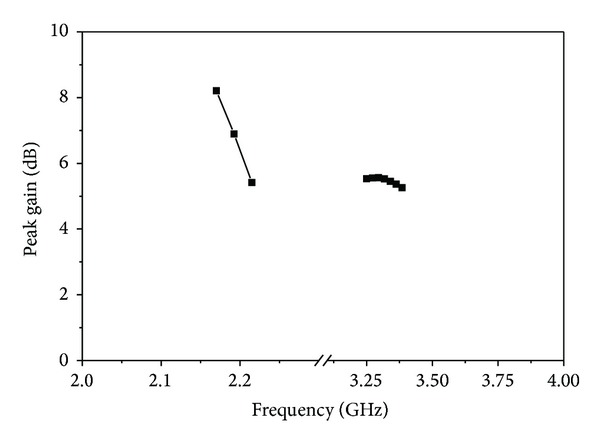
Measured gain versus frequency.

**Figure 14:**
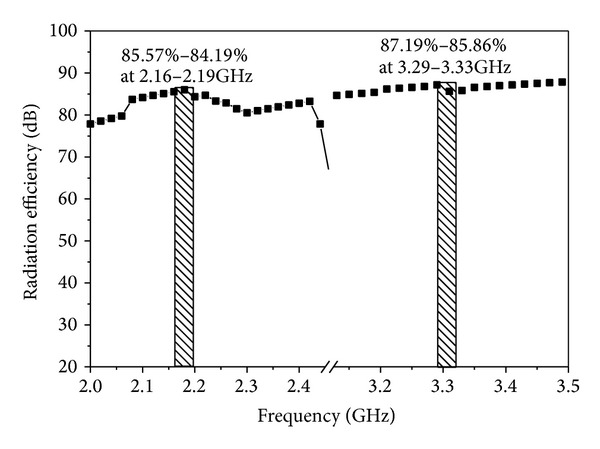
Radiation efficiency of the proposed antenna.

Figure 12 illustrates the measured radiation patterns for the proposed slotted antenna with optimized parameters in the XOZ plane at 2.18 GHz and 3.30 GHz, respectively. Actually, the printed slot antenna is a bidirectional radiator and the radiation patterns on both sides are the same.  Figure 13 shows the measured gain of the proposed antenna. The achieved gain of the lower band is defined in a right-hand circularly polarized source, while that for the upper band is left handed. The peak gains of the lower and upper bands are 6.59 dBiC and 5.52 dBiC, respectively. The radiation efficiency of the proposed antenna is painted in Figure 14. It is observed that the designed antenna achieved maximum radiation efficiency at lower band 85.79%–84.52% and upper band 87.79%–85.66%, respectively.

## 4. Conclusion

A single-feed circularly polarized dual frequency compact rectangular patch antenna is proposed and experimentally studied in this paper. A probe-fed square patch is used with a centre cross-shaped asymmetric slot for circular polarization and edge slots for dual frequency applications. 3D electromagnetic solver HFSS is used to optimize the initially designed antenna while maintaining a simpler design and lower profile than some other published structures. The proposed antenna is fabricated using a glass microfiber reinforced PTFE microwave substrate and found to have a S_11_ of −14.71 dB at 2.18 GHz and −18.72 dB at 3.30 GHz with 30 MHz and 40 MHz bandwidths, respectively. The peak gains are about 5.90–5.58 dBiC at lower band and 7.27–6.54 dBiC at higher band, respectively. It can be concluded that, since the proposed prototype is made of PTFE composite with dual frequency CP, this antenna can be applied to TT&C (Telemetry tracking and command) satellite applications.

## Figures and Tables

**Table 1:** Proposed antenna specification.

Parameter	(mm)
*L*	40.0
*W*	40.0
*L*1	16.0
*L*2	3.60
*L*3	2.80
*L*4	24.0
*t*	1.575
*L*5	5.0
*L*6	4.0
*a*	16.0
*b*	21.0
*d*	1.0
*d*1	1.0
*d*2	2.0

**Table 2:** Dielectric properties of substrate materials.

Substrate material	Relative permittivity (*∈*_*r*_)	Dielectric loss tangent	Relative permeability
Glass microfiber reinforced PTFE	2.33	00.0012	1
Epoxy resin-fibre glass	4.50	000.02	1
PTFE/woven-glass composite	2.20	0.0009	1
Taconic	06.50	0.0028	1
PTFE ceramic	10.20	0.0023	1
Neltec	2.08	0.0006	1
